# Cortical spreading depression can be triggered by sensory stimulation in primed wild type mouse brain: a mechanistic insight to migraine aura generation

**DOI:** 10.1186/s10194-022-01474-0

**Published:** 2022-08-19

**Authors:** Sahin Hanalioglu, Aslihan Taskiran-Sag, Hulya Karatas, Buket Donmez-Demir, Sinem Yilmaz-Ozcan, Emine Eren-Kocak, Yasemin Gursoy-Ozdemir, Turgay Dalkara

**Affiliations:** 1 Institute of Neurological Sciences and Psychiatry, Hacettepe University, Ankara, Turkey; 2 Department of Neurosurgery, Faculty of Medicine, Hacettepe University, Ankara, Turkey; 3 Present address: Department of Neurology, Faculty of Medicine, TOBB University of Economics and Technology, Ankara, Turkey; 4 Department of Psychiatry, Faculty of Medicine, Hacettepe University, Ankara, Turkey; 5 Present address: Department of Neurology, Faculty of Medicine, Koc University, Istanbul, Turkey

**Keywords:** Cortical spreading depression, Ouabain, Asante Potassium Green-4, Photic stimulation, Whisker stimulation, Migraine

## Abstract

**Background:**

Unlike the spontaneously appearing aura in migraineurs, experimentally, cortical spreading depression (CSD), the neurophysiological correlate of aura is induced by non-physiological stimuli. Consequently, neural mechanisms involved in spontaneous CSD generation, which may provide insight into how migraine starts in an otherwise healthy brain, remain largely unclear. We hypothesized that CSD can be physiologically induced by sensory stimulation in primed mouse brain.

**Methods:**

Cortex was made susceptible to CSD with partial inhibition of Na^+^/K^+^-ATPase by epidural application of a low concentration of Na^+^/K^+^-ATPase blocker ouabain, allowing longer than 30-min intervals between CSDs or by knocking-down α2 subunit of Na^+^/K^+^-ATPase, which is crucial for K^+^ and glutamate re-uptake, with shRNA. Stimulation-triggered CSDs and extracellular K^+^ changes were monitored in vivo electrophysiologically and a K^+^-sensitive fluoroprobe (IPG-4), respectively.

**Results:**

After priming with ouabain, photic stimulation significantly increased the CSD incidence compared with non-stimulated animals (44.0 vs. 4.9%, *p* < 0.001). Whisker stimulation also significantly increased the CSD incidence, albeit less effectively (14.9 vs. 2.4%, *p* = 0.02). Knocking-down Na^+^/K^+^-ATPase (50% decrease in mRNA) lowered the CSD threshold in all mice tested with KCl but triggered CSDs in 14.3% and 16.7% of mice with photic and whisker stimulation, respectively. Confirming Na^+^/K^+^-ATPase hypofunction, extracellular K^+^ significantly rose during sensory stimulation after ouabain or shRNA treatment unlike controls. In line with the higher CSD susceptibility observed, K^+^ rise was more prominent after ouabain. To gain insight to preventive mechanisms reducing the probability of stimulus-evoked CSDs, we applied an A1-receptor antagonist (DPCPX) to the occipital cortex, because adenosine formed during stimulation from ATP can reduce CSD susceptibility. DPCPX induced spontaneous CSDs but only small-DC shifts along with suppression of EEG spikes during photic stimulation, suggesting that the inhibition co-activated with sensory stimulation could limit CSD ignition when K^+^ uptake was not sufficiently suppressed as with ouabain.

**Conclusions:**

Normal brain is well protected against CSD generation. For CSD to be ignited under physiological conditions, priming and predisposing factors are required as seen in migraine patients. Intense sensory stimulation has potential to trigger CSD when co-existing conditions bring extracellular K^+^ and glutamate concentrations over CSD-ignition threshold and stimulation-evoked inhibitory mechanisms are overcome.

## Background

Cortical spreading depression (CSD) is regarded as the neurophysiological correlate of migraine aura and as a potential trigger for migraine headache [1]. CSD is a slowly propagating depolarization wave that spreads through the gray matter by depressing electrical activity. CSD is induced by various methods in experimental animals to study the pathophysiology of migraine aura and headache [1–3]. These methods involve direct stimulation of the exposed brain tissue with electrical, mechanical (pinprick) or chemical (e.g. KCl, glutamate) stimuli. Recently, optogenetic methods made a relatively non-invasive CSD induction possible [4]. Although CSDs induced by any of these methods share similar characteristics with spontaneously occurring CSDs and have been instrumental to investigate the electrophysiological characteristics of CSD, associated blood flow changes and headache generating mechanisms, the tools used are yet non-physiological unlike the conditions spontaneously generating CSDs (auras) in patients. Therefore, experimental methods that mimic the human condition more closely are needed to understand the mechanisms generating CSD in migraineurs’ normally functioning brain, a crucial but underexplored step in migraine pathophysiology. We hypothesized that CSD could be non-invasively evoked by intense sensory stimulation in the mouse brain because migraine headaches can be triggered by intense light or auditory stimulation as well as exercise [5–8]. Since these physiological triggers can initiate migraine aura and headache in genetically or hormonally primed migraineur brain but generally not in non-migraineurs, we tested the effect of intense sensory stimulation on the mouse brain made susceptible to CSD by partial inhibition of Na^+^/K^+^-ATPase with low concentrations of ouabain or by knocking-down its α2 subunit. Ouabain application to the brain tissue is well known to induce repetitive CSDs [9]. However, its sub-threshold doses can predispose to CSD generation without triggering CSDs similar to the familial hemiplegic migraine type 2 (FHM2) patients having haploinsufficiency of ATP1A2 gene that encodes α2 subunit of Na^+^/K^+^-ATPase [10]. These patients have a normal brain function but are prone to occasional hemiplegic migraine with aura attacks. Like patients with FHM2, the knock-in mice bearing the human FHM2 mutation do not develop spontaneous CSDs but has a lower threshold for CSD generation induced by electrical or KCl stimulation [11, 12]. We chose α2-Na^+^/K^+^-ATPase as a target because it plays a significant role in removing K^+^ and glutamate released during excitatory synaptic activity [13–15]. We have found that priming the visual cortex with a low concentration of ouabain increased the CSD ignition probability by 15 times with photic stimulation during CSD-free periods lasting at least 30 min. On the other hand, photic or whisker stimulation rarely elicited CSDs when the cortex was primed with α2-Na^+^/K^+^-ATPase knockdown although α2 knockdown consistently lowered the KCl-induced CSD threshold, suggesting that, with the protocols applied, ouabain was more effective than knockdown in inhibiting K^+^ clearance. We showed with fluorescent imaging that, ouabain yielded higher extracellular concentrations of K^+^ during sensory stimulation, which was possibly needed to offset the suppressive effect of feed forward inhibition (FFI) evoked by sensory stimulation, limiting evolution of stimulus-evoked depolarizations to CSD [16]. We also found that adenosine formed during synaptic activity could lower the CSD susceptibility [17]. A similar disparity between sensory stimulation- and KCl-evoked CSDs was observed with adenosine antagonism as well; A1 receptor antagonist DPCPX induced spontaneous CSDs but did not significantly increase the probability of stimulus-evoked CSDs possibly due to FFI concomitantly evoked, pointing to the possibility that stimulation-evoked inhibitory mechanisms that limit CSD generation might have underappreciated in studies using non-physiological CSD ignition methods.

## Methods

### Animals

All animal experiments were performed in accordance with relevant guidelines and regulations, and were approved by Hacettepe University Animal Experiments Ethics Committee (2012/53–01, 2017/05–2). A total of 134 male and female Swiss albino mice (25 to 35 g) were used. All mice were housed with ad libitum access to food and water under a fixed 12-h light/12-h dark cycle.

### Anesthesia

Mice were anesthetized with intraperitoneal injection of xylazine (10 mg/kg) and urethane (1.25 g/kg), which suppresses cortical excitability relatively less compared to commonly used anesthetics like isoflurane and ketamine [18, 19]. After maintaining an adequate depth of anesthesia, mice were positioned prone and fixed in stereotaxic frame (Digital Lab Standard Stereotaxic Frame, Stoelting, USA). In photic stimulation experiments, eyes were covered by lubricant gel and eyelids were closed with surgical clips to avoid corneal drying and enhance cortical sensitivity to light [20]. The heart rate and tissue oxygen saturation were continuously monitored by a pulse oximeter (V3304 Digital Table-Top Pulse Oxymeter, Nonin, USA) throughout all experiments. 100% oxygen mixed with room air (2 l/min) was delivered to the spontaneously breathing mice to avoid hypoxia. Rectal temperature was maintained at 37.0 ± 0.2 °C using a homeothermic blanket (Harvard Apparatus, UK) for the duration of the experiment. The depth of anesthesia was checked with toe/tail pinch and/or eye blink reflex at 10–15 min intervals and additional doses of anesthetics were injected when needed. Animals with persistent hemodynamic instability or hypoxia were excluded from the study.

### Surgery

A midline incision was made over the scalp to expose the skull. One or two small circular areas (diameter < 1 mm) were thinned with a high-speed drill (WPI, USA) to house the electrode tips over the cranium covering the right hemisphere; one at the anterior parietal and the other one at the frontal region. During drilling process, the skull was continuously irrigated with cold saline to prevent thermal injury to the underlying cortex. A cranial window (2 mm in diameter) was opened over the cranium covering either the visual cortex (for photic stimulation) or somatosensory barrel cortex (for whisker stimulation) without damaging the underlying dura mater. In a subset of experiments, a plastic cylindrical tube (inner diameter and height, 2 mm) was placed over the exposed intact dura and fixed to the skull by dental acrylic to encircle the cranial window. This chamber allowed topical administration of ouabain or the fluorescent potassium probe (Asante Potassium Green-4) as well as fluorescent imaging.

### Photic and whisker stimulation

A custom-made photic stimulator was used to illuminate both eyes. The frequency, intensity, duration and pattern of the stimulation were adjustable using a stimulus isolator (World Precision Instruments A385, USA) and a computer interface (LabChart, AD Instruments, Australia). 5-mm, 2 V, white mini-LED bulbs, which produce little heat emission, were used for stimulation. The LEDs were mounted on flexible arms that allowed optimal positioning of the LEDs a few millimeters away from the eyes of the mouse in stereotaxic frame. Light was directed toward the eyes. Whiskers were cut to 1 cm and automatically stimulated by using a custom-made apparatus. This device allowed stimulation of the whiskers unilaterally with a tip blunted watercolor brush (no:8) in vertical plane at an adjustable frequency (4 to 30 Hz) without touching common fur or other parts of the face of the mouse placed in the stereotaxic frame.

### Laser speckle contrast imaging

Cerebral blood flow (CBF) changes were detected with laser speckle contrast imaging as described before [21]. Briefly, the region of interest was illuminated with a 785-nm laser diode (Thorlabs DL7140-201, Thorlabs, USA) and imaged under 4X magnification by using a stereomicroscope (Nikon SMZ 1000, Nikon, Japan) and a charge-coupled device camera (Basler 602F, Basler Vision Technologies, Germany). Consecutive raw speckle images were acquired at 100 Hz (an image set) at 1-s intervals, processed by computing the speckle contrast using a sliding grid of 7 × 7 pixels, and averaged to improve the signal-to-noise ratio. Speckle contrast images were converted to images of correlation time values, which are inversely and linearly proportional to the mean blood velocity. Image J 1.42 software (NIH, USA) was used to compute and pseudo-color the relative blood flow changes in the cortex after sensory stimulation compared to baseline values.

### Epifluorescence microscopy

In a subset of experiments, fluorescent-tagged ouabain (BODIPY® FL Ouabain, Molecular Probes, USA) was topically applied over the dura by a cotton ball soaked with 5 μl of 0.1 mM ouabain in saline. Animals were sacrificed and the brains were removed after 60 min. 20 μm-thick coronal sections were obtained from the frozen brains. Sections were cover-slipped with Hoechst-33258 to delineate the tissue architecture by staining cell nuclei and, imaged under a fluorescence microscope using appropriate filter sets to assess the penetration and diffusion of ouabain into the brain tissue. In preliminary experiments (*n* = 3 mice), we also evaluated the distribution of fluorescent-tagged ouabain after intracerebroventricular (icv) administration (5 μL, 100 μM) to see whether this route could provide sufficient concentrations in the cortex. However, 45 min after intracerebroventricular administration, ouabain only accumulated in the vicinity (around 100 μm) of the ventricle and did not diffuse to cortex so, this route of administration was not preferred.

### Electrophysiology

To record the DC potentials, one or two Ag/AgCl pellet electrodes (E205, 1 mm diameter, Warner Instruments, USA) were placed and fixed over the thinned skull as described above. The electrode tips were covered with EEG electrode gel to enhance the electrical contact with the cranial bone. A disc-shaped Ag–AgCl ground electrode (E242, 4 mm diameter, Warner Instruments, USA) was placed between the neck muscles. The signals were digitized and acquired at 1 Hz sampling rate, displayed and analyzed by PowerLab 16/35 data acquisition system (AD Instruments).

### CSD induction with sensory stimulation

For investigating the temporal relationship between the photic stimulation and CSD induction, an intermittent stimulation protocol (on–off cycles) was preferred to avoid desensitization to light. Furthermore, the room was kept dark and the eyelids were closed starting with induction of anesthesia until the stimulation in order to increase light sensitivity of the visual cortex [20]. A low concentration (0.1 mM) of ouabain was topically applied over the visual cortex to prime it for CSD generation. Following at least a 30-min CSD-free silent period after the initial CSD(s) in these mice, the eyes were opened and 1-min on–off cycles of photic stimulation (8–12 Hz) were started until a CSD was evoked or for a maximum duration of 10 min (5 cycles). If no CSDs were evoked, then the same stimulation pattern was repeated 10 min after the last stimulus. In case of spontaneous CSD generation, the next stimulation was initiated after a 30-min silent period has passed. Eyes were closed again right after a CSD was evoked until the next stimulation or during the 10-min pauses between the photic stimulation epochs. The experiment was terminated in 5 mice (out of 22) if the experiment duration exceeded 4 h (*n* = 2) or repetitive CSDs were observed during 2-h recording without a 30-min silent period between them (*n* = 2) or the animal was hemodynamically unstable (*n* = 1). We excluded 2 animals because photic stimulation coincided with terminal depolarization. The effect of photic stimulation on a group of α2-Na^+^/K^+^-ATPase knockdown mice was also evaluated with the same protocol but without ouabain application. Whisker stimulation experiments were carried out with the same protocol to the one applied for photic stimulation. For these experiments, ouabain soaked cotton ball was placed over the somatosensory barrel cortex and only female mice were used to increase the CSD susceptibility because their CSD threshold is lower [22]. Contralateral whiskers were stimulated as detailed above. The effect of whisker stimulation on a group of α2-Na^+^/K^+^-ATPase knockdown mice was also evaluated with the same protocol but without ouabain application.

### α2-Na^+^/K^+^-ATPase knockdown

pLL 3.7 plasmid expressing α2-Na^+^/K^+^-ATPase-shRNA was kindly provided by Dr. Gilbert Gallardo (Washington University, School of Medicine). α2-Na^+^/K^+^-ATPase-shRNA consists of 21-nucleotide inverse repeats (sense sequence: 5'-GTG GCA AGA AGA AAC AGA AAC-3') separated by a 9-nucleotide loop sequence (CAAGTTAAC). The shRNA construct in the vector was verified before use by sequencing. We used empty pLL 3.7 plasmid as a control. A total of 24 mice injected with shRNA and 15 mice with control blank plasmid. α2-Na^+^/K^+^-ATPase-shRNA expressing plasmid or control plasmid (1 μg) was mixed with 0.12 μl in vivo-JetPEI-TM (Polyplus, France) transfection reagent. Then 1 μl of this mixture was injected at two different points intracortically at a rate of 0.1 μl/min under isoflurane anesthesia by a 26 gauge Hamilton syringe. For this, the needle was lowered 1 mm deep and, after waiting for 1-min, it was slowly removed over 5 min to allow diffusion of the plasmids into cortical layers. The sites of injection in the CSD threshold (*n* = 6/group) and photic stimulation experiments were in the right visual cortex; (-3, 1.5) mm and (-3, 3.5) mm relative to bregma (anteroposterior and lateral coordinates, respectively) (*n* = 6). In whisker stimulation and K^+^ fluoroprobe imaging experiments, the sites of injection were in the right barrel cortex; (-0.5, 3.5) mm and (-1.8, 3) mm relative to bregma (*n* = 6). To verify knockdown with qRT-PCR, mice were sacrificed at 6, 24 and 48 h after shRNA delivery. The injection areas and the contralateral homologous areas were removed and RNAs were extracted with RNeasy Mini Kit (QIAGEN, GERMANY, cat no: 74104) according to instructions. Eluted RNAs were stored at -80 °C. cDNA synthesis was performed with random hexamer primers with RevertAid First Strand cDNA Synthesis Kit (Thermo Fisher Scientific, USA cat no: K1621) according to instructions. cDNAs were stored at -20 °C. qRT-PCR was performed with Taqman probe-based technology. Taqman gene expression master mix (ABI, USA cat no: 4369016), FAM-MGB labeled Taqman probes for mouse ATP1A2 gene (Assay ID: Mm00617899_m1) and mouse GAPDH gene (Assay ID: Mm9999991) were used. cDNAs were diluted to 1:32 ratio in nuclease free water. The PCR was carried out in triplicates in ABI OneStep Q RT PCR machine (ABI, USA). The relative expression values were calculated with ΔΔCt method.

### Detection of CSD threshold

To investigate the CSD threshold after knockdown, plasmid injected animals were tested either at 6 h, 24 h, or 48 h after injection. Increasing concentrations of KCl-adsorbed cotton balls were consecutively applied epidurally through the burr hole over the parietal bone. Each cotton ball was allowed to stay for 5 min. The DC potential was continuously recorded over the right visual cortex and the cotton ball was replaced with the next higher concentration if no CSD wave was observed during 5 min. Concentrations of KCl starting from 0.05 M and increasing by 0.025 M at each step were used to detect the threshold. Once CSD was induced, the experiment was ended. The minimum concentration that yielded the first CSD was accepted as the threshold.

### Monitoring extracellular K^+^ in vivo with a fluoroprobe

In order to investigate the extracellular potassium changes, a fluorescent potassium probe, Asante Potassium Green-4 (IPG-4, formerly APG-4, TefLABs, USA) was used. IPG-4 has a higher selectivity for K^+^ over Na^+^ than previous Asante potassium dyes used in vivo [23].

IPG-4 was prepared in 2% DMSO and diluted in artificial cerebrospinal fluid (aCSF) to yield a final concentration of 250 μM. Since it was poorly soluble in aqueous media, ultrasonic bath and gentle heating was applied to increase its solubility on the day of each experiment. Before using for stimulation experiments, the potassium selectivity of IPG-4 was characterized in order to assess its compatibility with the large ionic shifts during CSD. For this, we applied three different concentrations of K^+^ (0, 10, 50 mM) in the presence of high Na^+^ concentrations in distilled water. Fluorescence intensity showed gradual increase with rising concentrations of K^+^ and was similar in the presence of 77 mM or 154 mM Na^+^ in the medium. Next, we investigated the distribution of IPG-4 in the mouse brain following icv (*n* = 2) injection or topical epidural application (*n* = 3) under an upright fluorescent microscope. Following icv injection, dye was largely accumulated in the periventricular area and did not reach the cortex. However, after topical application through a chamber over the exposed dura, IPG-4 diffused into superficial cortical layers down to 200 μm, which was satisfactory because fluorescence microscopy is limited to image only the superficial 50–70 μm of the cortex in vivo. Therefore, the epidural application was used for intravital imaging of extracellular potassium dynamics in the cortex. To detect the extracellular K^+^ change in vivo, a baseline image was captured for obtaining autofluorescence intensity of the tissue and the closed cranial chamber was filled with 8–10 μl of 250 μM IPG-4. The room was darkened during 30-min dye incubation. Then the dye was removed and the chamber was gently rinsed with aCSF three times at 37 °C. Chamber was filled completely with aCSF to avoid any air bubble formation and closed by a clean cover glass for optimal fluorescence microscopy. The images were acquired under a Nikon SMZ 1000 stereomicroscope with a fluorescent attachment by using a CCD camera (Nikon DS-Qi1Mc, Japan) and NIS Elements Advanced Research Software v3.2. We used a fluorescence filter (HQ FITC LP) with excitation: 480/40 and emission: > 510 nm, an exposure time of 250 ms and a signal averaging of 4x for acquisition. Each imaging session lasted 7 min. Time-lapse recording for every 5 s started 1 min before whisker stimulation, continued through the 5 min of stimulation and ended 1 min after.

For the analysis of IPG-4 fluorescence, an ROI was placed in a predefined area that showed the maximum hyperemic response with whisker stimulation in preliminary experiments. The mean fluorescent intensity of the baseline image was subtracted from the time series images. A rolling average by 10 was applied to the image sequence. By using the *time measurement* feature of Nikon NIS-AR software, we acquired a set of signal intensity values for each session of whisker stimulation. This data set was exported for further processing and calculations to MATLAB (Mathworks, USA) software. In MATLAB (Mathworks, USA), the baseline drift that frequently accompanied the IPG-4 fluorescence was corrected by *detrending* and then the percent change from baseline (dF/F_0_%) was calculated. To incorporate both the duration and the amplitude of stimulation-induced signal change into analysis, we calculated area-under-curve (AUC) and compared AUC between groups.

### Chemicals

Ouabain octahidrate (Sigma-Aldrich, USA), a Na^+^/K^+^-ATPase inhibitor, was topically applied with a micropipette at a total volume of 5 μL in saline either onto the cotton ball placed over the intact dura or, for IPG-4 experiments, into the cranial window 30 min before IPG-4 incubation. For assessing the incidence of sensory stimulation-induced CSDs, electrophysiological recordings were started before ouabain applications and continued 150 min to monitor CSD occurrence. To determine the threshold dose for CSD induction, ouabain solutions at different concentrations (0.05, 0.1, 0.15, 0.3, 0.5 mM) were tested. The time to CSD induction and CSD frequency were measured for each tested concentration in a fixed volume (5 μl). 8-Cyclopentyl-1,3-dipropylxanthine (DPCPX, Tocris, UK), a high affinity adenosine A1 receptor selective antagonist, was dissolved in DMSO as a stock solution (30 mM). On the day of each experiment, the drug was suspended in saline and gently heated at a concentration of 30 μM for intracortical injection or 0.7–1 mM for epidural application with a cotton ball.

### Statistics

Data were presented as mean ± SE or median (range) or percentage (%) of the total and were analyzed using chi-square test or Mann–Whitney U test, where appropriate. To differentiate sensory stimulation induced CSDs from spontaneously occurring CSDs, a modified version of the method previously used by von Bornstadt et al. was adopted [24]. Briefly, we divided all the time-series monitoring data into 10-min (photic) or 5-min (whisker) epochs. We then assessed whether any epoch has CSD. If an epoch has CSD, it gets “1”, otherwise “0”. Cumulative incidences for groups were then calculated and compared with Chi-square test.

## Results

### Detecting around-the-threshold concentration of ouabain for CSD induction

We were not able to induce CSD by photic stimulation applied with various parameters in preliminary experiments in both sexes of mice. Therefore, we searched an ouabain dose that does not cause continuous repetitive CSDs but primes the cortex near to the CSD threshold to be able to test any CSD generating potential of sensory stimulation. No CSD was induced with 0.01 and 0.05 mM concentrations of ouabain topically applied over the cortex for 150 min, whereas repetitive CSDs were evoked (> 5 CSDs within 150 min) with 0.3 mM (*n* = 4) or 1 mM (*n* = 4) in every animal tested (Table 1). With 0.1 mM ouabain (*n* = 16), no CSD was evoked in 25%, 1–2 CSDs in 50% (38 and 12%, respectively) and > 5 CSDs were detected within 150 min (i.e. ≥ 3 CSDs per hour) in only 25% of the experiments, whereas more than 5 CSDs were recorded in 52% of experiments with 0.15 mM (*n* = 25) (Table 1). The mean latency to the first CSD was short with 0.3 and 1 mM ouabain (21 ± 5.6 and 13 ± 2.5 min), in contrast, it was significantly delayed with 0.1 mM (46 ± 4.4 min) concentration (*p* = 0.001) (Table 1). Therefore, 0.1 mM ouabain was chosen for creating susceptibility to CSD in sensory stimulation experiments because the probability of spontaneous CSD induction was low and, a long-enough silent period after the initial CSDs allowed testing the effect of stimulation on a primed cortex. Indeed, in experiments in which sensory stimulation was effective in inducing CSD (14 out of 26 mice), 0.1 mM ouabain triggered at least one initial “spontaneous” CSD (7 out of 14 mice) or 2 (3 mice), 3 (1 mouse), 7 (2 mice) and 13 CSDs (1 mouse) before the silent period during which stimulation effect was tested. In line with the dose screening experiments without sensory stimulation, in only 21% of the mice (3 out of 14) more than 3 spontaneous CSDs occurred before entering the silent period. We waited to see the initial CSDs to ensure that the cortex was primed, which subsequently remained silent at least 30 min before recurrence of another CSD (if any), allowing assessment of the stimulation effect. We named 0.1 mM concentration "around-the-threshold" because the initial supra-threshold tissue concentration becomes sub (near)-threshold over time, creating silent periods of at least 30 min and, seldom re-passes the threshold, igniting non-stimulated CSDs after last non-stimulated or stimulated CSD (observed in only 1 out of 14 animals). This was consistent with both concentration optimization experiments (no stimulation, *n* = 16 mice) and sensory stimulation experiments (14 mice). This allowed us to calculate the probability of a non-stimulated CSD occurrence for any given time frame after 30 min of silent period, and compare it with that of stimulated CSDs.

**Table 1:** CSD onset latency and frequency with different concentrations of topical ouabain application in the mouse brain (total volume for each animal equals to 5 μl in all experiments). (none: no CSD detected, rare: 1–2 CSDs/h, frequent: ≥ 3 CSDs/h)

Concentration (mM)	Latency to CSD onset (min) (mean ± SE)	Latency to CSD onset (min) (range)	CSD pattern (frequency)
0.1	46.3 ± 4.4	6—68	% 25 none % 50 rare % 25 frequent
0.15	40.2 ± 4.2	14—68	% 20 none % 28 rare % 52 frequent
0.3	20.7 ± 5.6	6—33	%100 frequent
1	13.2 ± 2.5	7—14	%100 frequent

### Optimization of photic and whisker stimulation

In order to determine the optimal sensory stimulation parameters and accurate mapping of respective sensory cortices, regional CBF changes were measured following photic or whisker stimulations on occipital and barrel cortices, respectively. Maximal CBF changes at visual cortex were observed with 8–12 Hz photic stimulation in preliminary experiments (*n* = 3 mice). The peak increase in CBF at these frequencies was 11.9 ± 2.1%, whereas the mean CBF change during the stimulation period was 7.6 ± 1.7%. The CBF increase reached a maximum within 15–30 s followed by a plateau during the stimulation and rapid decline to baseline after the stimulation. The slope of the plateau was about zero for 1-min stimulation period while it turned negative in longer duration of stimulations, possibly representing adaptation of visual cortex to continuous stimulation. Accordingly, 1-min stimulation period was chosen for the visual stimulation experiments. Maximal CBF changes at the somatosensory barrel cortex were observed with whisker stimulation at 12–20 Hz. The peak CBF change for 12 Hz frequency was 12.4 ± 3.3%, whereas the mean CBF change during stimulation period was 5.1 ± 1.4% (*n* = 3 mice). The CBF increase reached a maximum within 30–60 s followed by a plateau during the stimulation and declined to baseline within 15–20 s after the stimulation. The slope of the plateau was about zero up to 10-min stimulation periods. Accordingly, whisker stimulation was continued for 5 and occasionally 10 min.

### CSD induction with photic stimulation in pharmacologically primed mouse visual cortex

**fig. 1:**
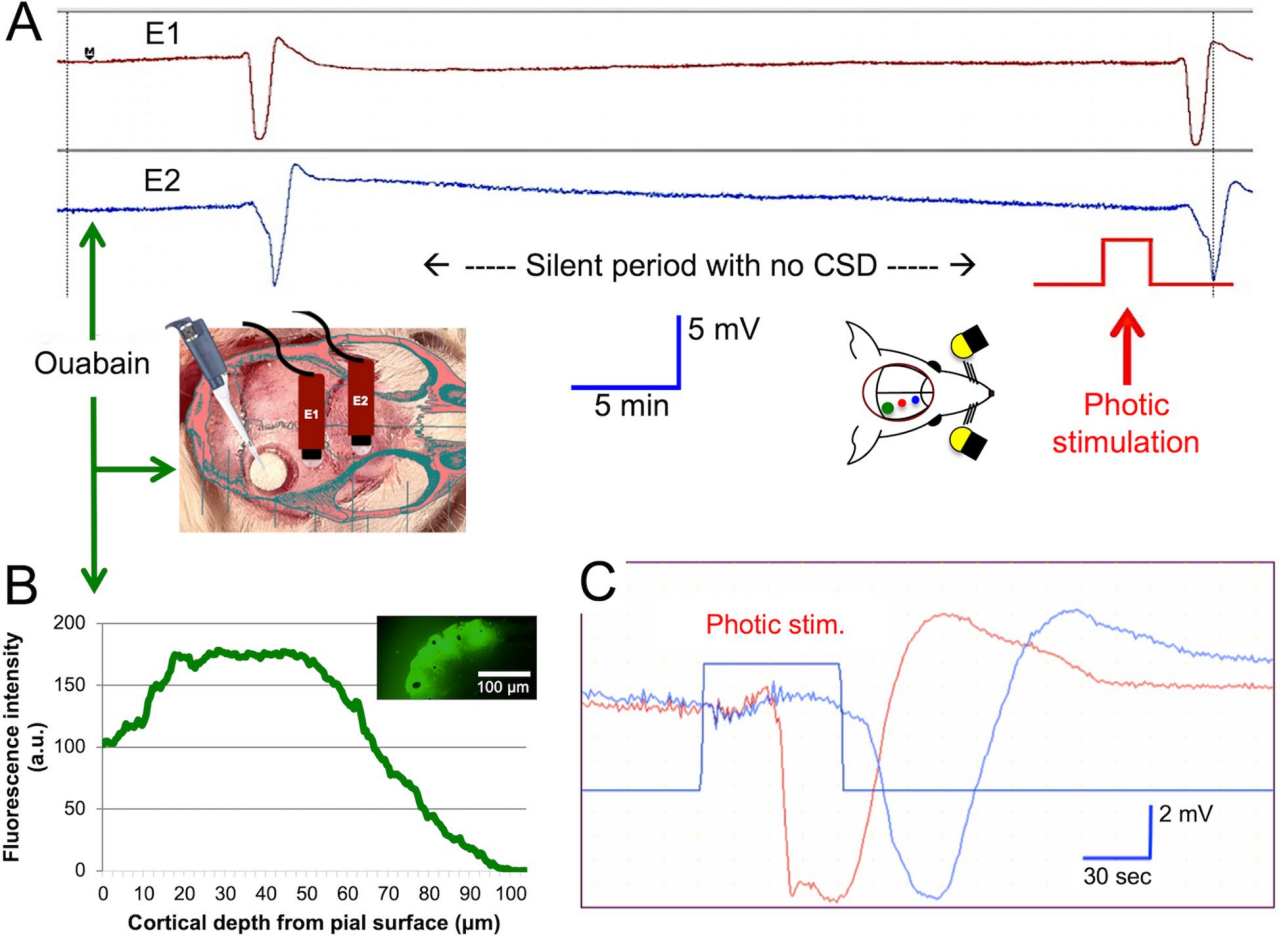
Photic stimulation evokes CSD in the visual cortex primed with around-the threshold dose of ouabain. A shows the DC potential recordings from 2 extracranial electrodes placed 1 mm apart anterior to the cranial window opened over the occipital cortex. Ouabain (0.1 mM) application over the dura induced a single CSD. After a silent period of 30 min without any CSD, photic stimulation (1 min at 12 Hz) evoked a CSD in this mouse. Below schematics illustrate the placement of electrodes and cranial window over the mouse skull and photic stimulation setup (right). B illustrates that penetration of the fluorescent-labeled ouabain (BODIPY 0.1 mM, epidural) is limited to the superficial layers of the cortex. C shows another example of CSD evoked with photic stimulation. The distance between CSD generating primed occipital cortex area and recording electrodes causes the latency between the photic stimulation pulse and DC shifts

**fig. 2:**
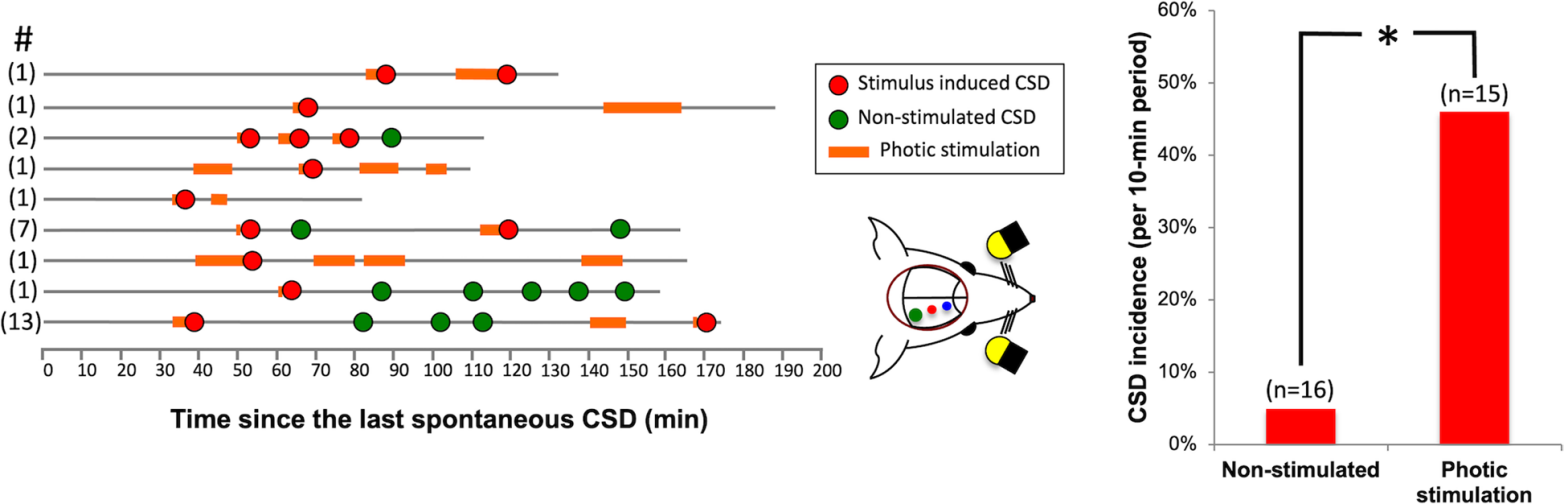
Priming the occipital cortex with ouabain increased the incidence of photic stimulation-induced CSDs. Each horizontal line represents continuous recording from one mouse primed with 0.1 mM ouabain and then subjected to 1-min on and off cycles of photic stimulation at 8–12 Hz for 10 min except a few that lasted a bit shorter or longer due to interventions needed (e.g. for clearing the airway) or timing errors in stimulator. Stimulation started after at least a 30-min silent period without any CSD. The number of ouabain-induced CSDs prior to starting stimulation is given before each line in parentheses. Stimulation (labeled orange) was stopped when a CSD was triggered (red circles). Green circles depict the occasional CSDs unrelated to stimulation. Bar graph shows that the CSD incidence was significantly higher (*p < 0.001, Chi-square test) during photic stimulation compared to the non-stimulated periods. Only positive experiments are displayed on the left to simplify viewing of the flow charts by omitting the negative experiments (*n* = 6), but statistical analysis on the right included all animals regardless of the stimulation effectiveness (15 mice) and compared them to 16 non-stimulated mice treated with 0.1 mM ouabain. Because the CSD incidence was very low in controls, it was not suitable for illustration (i.e. 16 lines mostly with no incidences)

With intermittent photic stimulation of the ouabain-primed visual cortex, a strong probable relationship was found between the photic stimulation and CSD occurrence in 9 of 15 animals tested (60%)(Fig. 1, 2). A total of 16 CSDs were recorded during or just after 1-min photic stimulation pulse following at least a 30-min CSD-free “silent” period with 0.1 mM ouabain. The time elapsed between the start of photic stimulation cycles and the CSD generation was 4.1 ± 1.0 min on average but was quite variable ranging from 20 secs (i.e. with the first pulse of stimulation) to 10 min (i.e. with the last 1-min pulse). To prove that the observed CSDs were indeed triggered by photic stimulation, we tested whether or not the incidence of CSDs following photic stimulation differs from that of the spontaneously emerging (“non-stimulated”) CSDs after priming with around-the-threshold (0.1 mM) concentration of ouabain but without photic stimulation. A probabilistic approach previously used by von Bornstadt et al.[24] was adopted to test this possibility. The average non-stimulated CSD incidence calculated by measuring the chance of CSD occurrence for each 10-min epochs (total stimulus duration) following a 30-min silent phase was found to be 4.8%. However, the average incidence of CSDs after photic stimulation was 44%, which was significantly higher compared to the stimulus-independent CSD incidence (OR = 14.8, 95% CI: 3 – 72, *p* < 0.001).

### CSD induction with whisker stimulation in pharmacologically primed mouse barrel cortex

**fig. 3:**
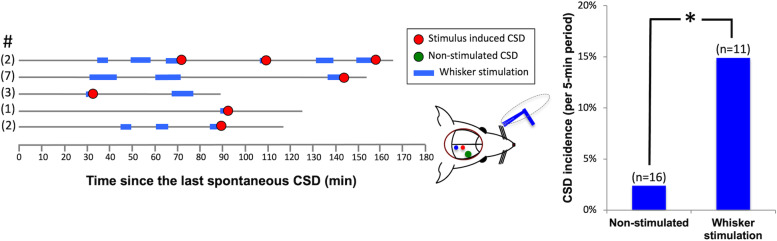
Priming the barrel cortex with ouabain increased the incidence of whisker stimulation-induced CSDs. Each horizontal line represents continuous recording from one mouse primed with 0.1 mM ouabain and then subjected to whisker stimulation at 8–12 Hz for 10 min except a few that lasted a bit shorter or longer due to interventions needed (e.g. for clearing the airway) or timing errors in stimulator. Stimulation started after at least a 30-min silent period without any CSD. The number of ouabain-induced CSDs prior to starting stimulation is given before each line in parentheses. Stimulation (labeled blue) was stopped when a CSD was triggered (red circles). Green circles depict the occasional CSDs unrelated to stimulation. Bar graph shows that the CSD incidence was significantly higher (*p = 0.026, Chi-square test) during whisker stimulation compared to the non-stimulated periods. Only positive experiments are displayed on the left to simplify viewing of the flow charts by omitting the negative experiments (*n* = 6), but statistical analysis on the right included all animals regardless of the stimulation effectiveness (11 mice) and compared them to 16 non-stimulated mice treated with 0.1 mM ouabain. Because the CSD incidence was very low in controls, it was not suitable for illustration (i.e. 16 lines mostly with no incidences)

We extended our study to include whisker stimulation based on a report suggesting that whisker cortex in the mouse has a lower CSD threshold compared to other cortical regions [25]. We hypothesized that this regional susceptibility in the mouse cortex could increase the sensory stimulus-evoked CSD probability. In a different set of animals, a similar experimental protocol resulted in CSD induction following whisker stimulation in 5 of 11 mice tested (45.5%)(Fig. 3). A total of 7 CSDs associated with stimulation were recorded in those 5 animals. The duration between the start of whisker stimulation and the CSD generation was 2.8 ± 0.6 min on average but was quite variable ranging from 20 s (i.e. at the beginning of the first 5-min pulse) to 8 min (i.e. during the second pulse). Average incidence of CSDs for each 5-min periods corresponding to duration of whisker stimulation was 14.9%, while it was only 2.4% for spontaneous CSDs emerging without whisker stimulation (OR = 7, 95% CI: 1.4 – 35.2, *p* = 0.02). In conclusion, although whisker stimulation has potential to trigger CSDs, it was less efficient compared to photic stimulation.

### Distribution of BODIPY-ouabain in the mouse brain

To evaluate the distribution of ouabain in the mouse cortex after focal epidural administration, we applied fluorescent-labeled ouabain (BODIPY-ouabain) at the same dose (5 μl, 0.1 mM) and manner as used in priming experiments. Mice (*n* = 3) were sacrificed 45 min after the drug administration and brain sections were evaluated under fluorescence microscope. Ouabain was largely accumulated in dura mater and the underlying superficial cortical layers. The signal intensity steeply declined as the drug penetrated into deep cortical layers (Fig. 1B). Beyond 140.6 ± 13.4 μm depth, fluorescent signal was barely detectable (< 1% of maximum intensity detected in more superficial layers). Longitudinally, fluorescence signal was restricted to cranial window area with no significant lateral spread beyond its borders.

### *Susceptibility to CSD after partially silencing α2-Na*^+^*/K*^+^*-ATPase expression*

**fig. 4:**
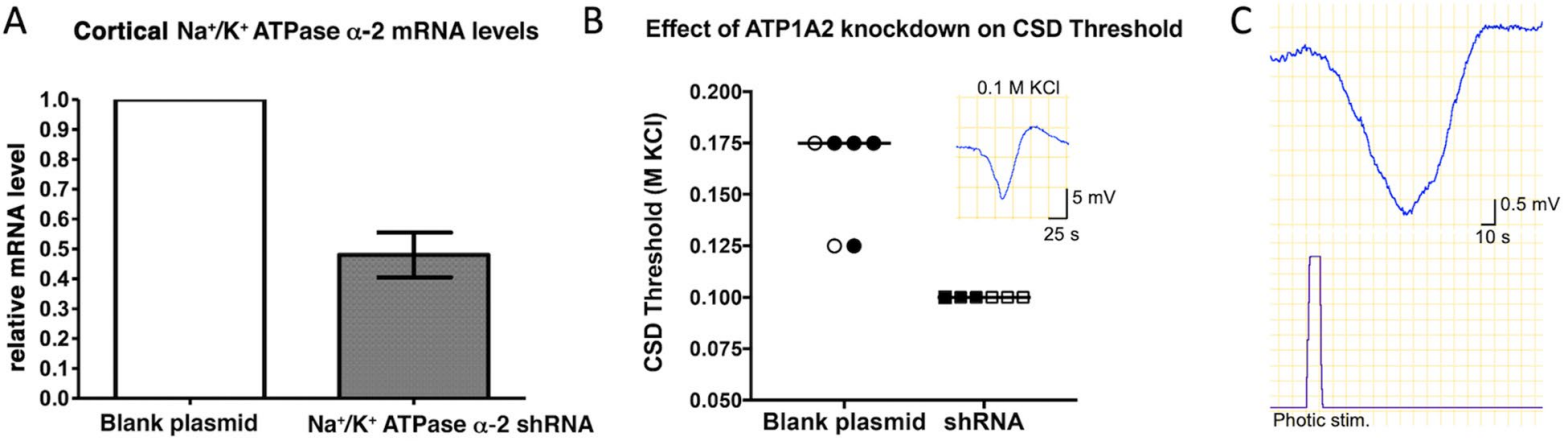
Knocking down cortical α2-Na^+^/K^+^-ATPase lowered the CSD threshold. Intracortical injection of α2-Na^+^/K^+^-ATPase shRNA-coding plasmids to the occipital cortex led to a 50% decrease in α2-Na^+^/K^+^-ATPase mRNA level as detected by qRT-PCR (left, *p* = 0.07, unpaired t-test) and reduced the CSD induction threshold (right) as assessed with varying concentrations of KCl (from 0.050 to 0,175 M) 6 (clear symbols) or 24 h (filled symbols) after injection compared to the blank plasmid-injected group (median = 0.1 M KCl vs 0.175 M KCl, *p* = 0.002, Mann–Whitney U test). A representative KCl-triggered CSD recorded from an ATP1A2 knockdown cortex is shown over the shRNA data points. A photic stimulation-triggered CSD (without KCl or ouabain treatment) from an ATP1A2 knockdown mouse is displayed on the right

We validated α2-Na^+^/K^+^-ATPase knockdown by qRT-PCR and found a 50% decrease in α2-Na^+^/K^+^-ATPase mRNA levels in the occipital cortex injected with α2-Na^+^/K^+^-ATPase-shRNA compared to the control group (*n* = 3 mice/group) (Fig. 4). Intracortical knockdown of α2-Na^+^/K^+^-ATPase led to a decrease in the CSD threshold at 6 (*n* = 3) and 24 (*n* = 3) hours following injection of the plasmid (median = 0.1 M KCl vs. 0.175 M KCl in α2-Na^+^/K^+^-ATPase-shRNA injected and blank plasmid injected controls (*n* = 6), respectively; *p* = 0.002)(Fig. 4). No spontaneous CSDs were observed in these mice during 8 ± 2 min monitoring before induction with KCl. In another set of α2-Na^+^/K^+^-ATPase knockdown mice, photic stimulation induced CSDs in 1 out of 7 mice 24 h after injection, whereas whisker stimulation triggered CSDs in 1 out of 6 mice. Two additional α2-Na^+^/K^+^-ATPase knockdown mice were recurrently tested with photic cycles at 6 h, 24 h and 48 h (under brief isoflurane anesthesia on each day) not to miss any effective time point; however, we did not observe CSD at any time point, altogether suggesting that the probability of CSD induction by sensory stimulation is low after partial α2-Na^+^/K^+^-ATPase knockdown despite reduced KCl-induced CSD threshold in all of these mice.

### Extracellular potassium changes during sensory stimulation

**fig. 5:**
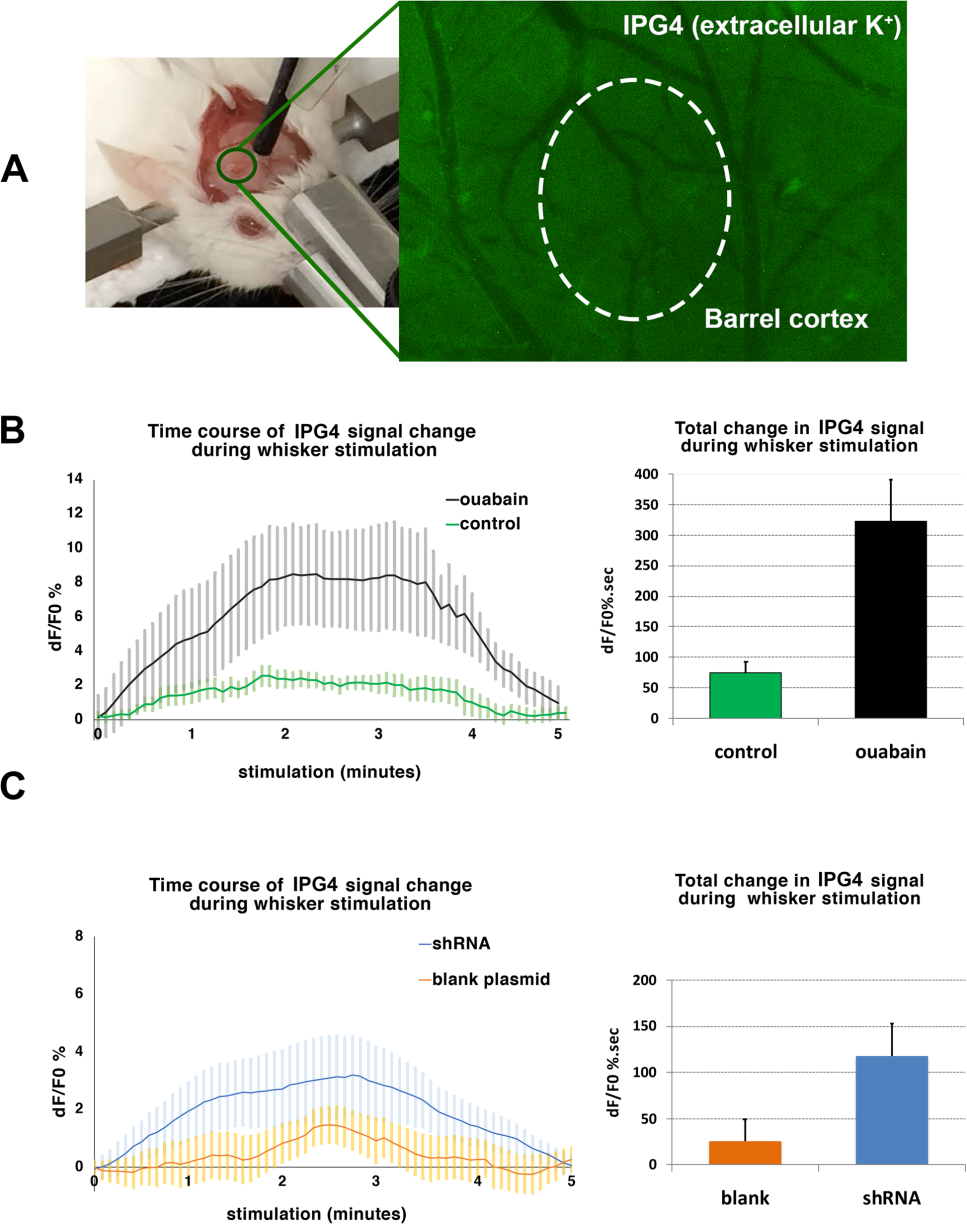
α2-Na^+^/K^+^-ATPase knockdown or ouabain reduced K^+^ uptake during whisker stimulation. In order to investigate the extracellular potassium changes, a fluorescent potassium probe, Asante Potassium Green-4 (IPG-4) was used. A The potassium sensitivity of IPG-4 fluorescence was linear within the range encountered in the extracellular space during CSD as assessed in vitro. B and C We monitored the extracellular potassium changes through a cranial window placed over the barrel cortex (picture) by plotting the relative fluorescence intensity change (dF/F0) over time (graphs on the left) and calculating the area under the curve (bars on the right). In ouabain-primed (0.1 mM) cortex, 5 min of 10 Hz whisker stimulation resulted in a significant increase in potassium fluorescence compared to aCSF-treated control group (*n* = 6 mice for each group)(*p* = 0.004, Mann–Whitney U test). Similarly, there was significant increase in potassium signal during whisker stimulation in α2-Na^+^/K^+^-ATPase shRNA-encoding plasmid injected mice (*n* = 5) compared to blank plasmid injected animals (*n* = 6)(*p* = 0.055, Mann–Whitney U test). Error bars represent SEM

To assess whether or not ouabain or α2-Na^+^/K^+^-ATPase knockdown did effectively reduce K^+^ uptake, we monitored the extracellular potassium change during intense whisker stimulation by plotting the relative fluorescence intensity change (dF/F_0_) over time and calculating the area under the curve (AUC). In ouabain-primed (0.1 mM) mice, 5 min of 10 Hz whisker stimulation resulted in a significant increase in K^+^ fluorescence compared to aCSF-treated control group (*n* = 6 mice for each group, 323 ± 68 vs. 74 ± 19 dF/F_0_%.sec, *p* = 0.004) (Fig. 5A). Similarly, there was significant increase in potassium signal during whisker stimulation in α2-Na^+^/K^+^-ATPase shRNA-encoding plasmid injected mice (*n* = 5) compared to blank plasmid injected animals (*n* = 6)(118 ± 35 vs. 25 ± 24 dF/F_0_%.sec, *p* = 0.055), however, to a notably lesser degree than ouabain induced rise in line with higher probability of sensory stimulation-induced CSDs in ouabain-primed mice (Fig. 5B). Inhibition of α3 isoform in addition to stronger α2 suppression by ouabain can account for this relatively larger K^+^ surges seen after ouabain [26]. Moreover, the plasmid injection-induced cortical injury might have mildly suppressed whisker stimulation-evoked neural activity (hence, the K^+^ release as seen in the blank plasmid group compared to controls; Fig. 5C). An acute but moderate suppression of the neural activity is documented after cranial window surgery and may reportedly last for a few weeks [27]. Whisker stimulation induced CSDs in 1 out of 6 knockdown mice as indicated above, which was excluded from the K^+^ signal analysis because a marked increase in fluorescent intensity coincident with an electrophysiologically recorded DC shift was observed. The increased K^+^ signal propagated at a speed of 1.5 mm/min in accordance with a spreading depolarization.

### Breaking mechanisms reduce CSD incidence

**fig. 6:**
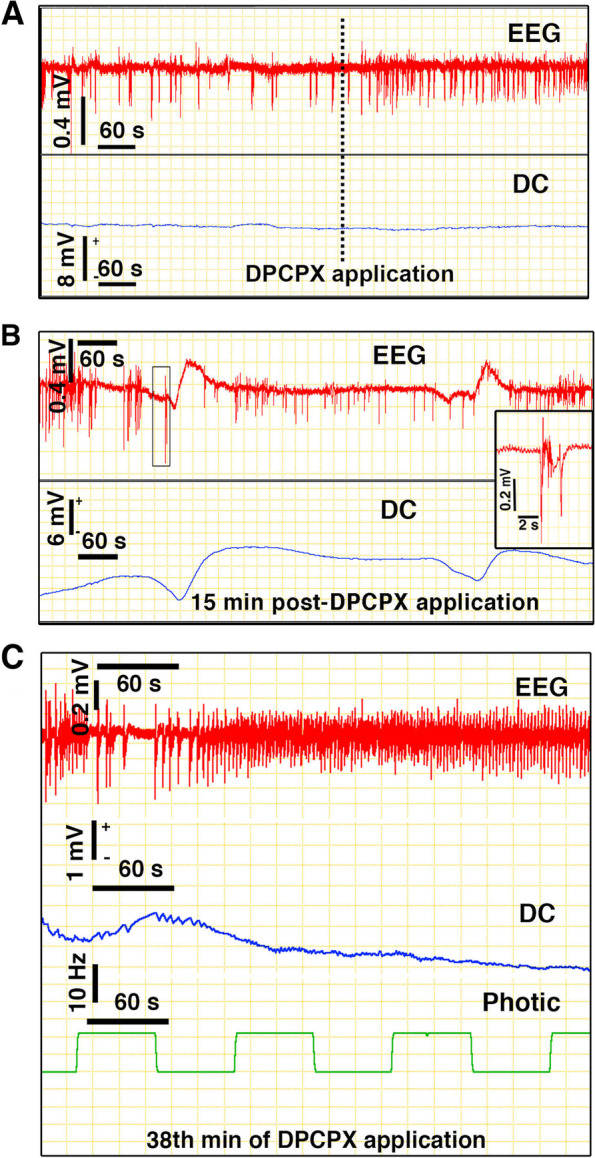
Adenosine generated during synaptic activity may be one of the safety mechanisms preventing CSD ignition. A Epidurally applied selective A1-receptor antagonist, DPCPX (1 mM) induced irregularly spaced synchronized discharges, indicating that it sufficiently penetrated cortex. B DPCPX applied to the non-primed occipital cortex caused full-blown CSDs without stimulation in 4 out of 8 mice, whereas photic stimulation evoked a small-DC shift during which ongoing EEG activity (except large-amplitude synchronized discharges) was suppressed in 10 out of 116 photic stimulation periods (**C**). EEG recordings were obtained with extracranial electrodes. Magnified EEG shown at an expanded time scale in inset in B disclosed high-amplitude discharges just before CSDs, possibly corresponding the neuronal synchronization preceding CSD generation

Priming the mouse brain by targeting Na^+^/K^+^-ATPase increased the probability of CSD generation with sensory stimulation. However, its probabilistic nature suggested presence of braking mechanisms that prevent full-blown CSD ignition. We hypothesized that adenosine generated during synaptic activity by breakdown of ATP could decrease glutamate release from thalamocortical nerve endings and suppress post-synaptic excitability, hence, counteract the depolarizing drive for CSD ignition [17]. This can be one of the several safety mechanisms that prevent CSDs in the normal brain. To test this hypothesis, we used a selective A1-receptor antagonist (DPCPX) to reduce the adenosinergic inhibition on glutamate release and postsynaptic excitability [28, 29]. DPCPX was applied to the non-primed occipital cortex epidurally (0.7–1 mM) in 5 mice and intracortically (30 μM, 200 μm deep) [17] in 3 additional mice without any notable differences in the results. In 4 of these mice, DPCPX application over dura caused 6 full-blown CSDs before stimulation (Fig. 6B). When DPCPX concentration was below the threshold to ignite full-blown spontaneous CSDs, photic stimulation (116 sessions in 6 mice) induced 10 small-DC shifts (coincident with intermittent suppression of EEG spiking) with mean amplitude of 1.4 ± 0.2 mV and duration at half maximum of 90 ± 17 s (Fig. 6C). Of note, since the recordings were made over the skull without opening a burr hole to maintain the cortical physiology intact at the recording site, the amplitudes are smaller compared to the intracortically recorded CSDs. So, we normalized the amplitude of small-DC shifts to the pinprick-induced full-blown CSDs at the end of the experiment, which was 17.3 ± 1.8% to assess their magnitude relative to full-blown CSDs. Because of the poor solubility of DPCPX like most other A1 antagonists and rapid absorption of its solvent DMSO through the dura underlying the cranial window during prolonged application, a precise control over the final tissue concentration achieved in cortex was not possible, hence, combination experiments with ouabain or dose–response experiments for detecting the thresholds of full-blown CSD and small-DC shift were not feasible. However, on topical application, we ensured that DPCPX sufficiently penetrated cortex by observing appearance of irregularly spaced large-amplitude (synchronized) EEG discharges (Fig. 6A). We think that the DC shifts and large-amplitude EEG spikes observed during photic stimulation may reflect the intense dendritic depolarizations potentiated with DPCPX; whereas the intermittent suppression of EEG spikes, the oscillations induced by the stimulus-triggered inhibitory (and excitatory) activity (Fig. 6C). This is not surprising because DPCPX can potentiate the glutamatergic synaptic activity terminating on inhibitory neurons as well [16], thus, preventing progress of the enhanced excitatory input on principal neurons to full blown CSDs.

## Discussion

In this study, we have shown that intense sensory stimulation, one of the triggers inducing migraine with aura, has indeed potential to ignite CSD in mouse brain primed by promoting extracellular K^+^ and glutamate concentrations with pharmacological or genetic means. The priming with a low ouabain concentration (0.1 mM) increased the chance of CSD triggering with light flashes as high as 15 times compared to non-stimulated ouabain-treated controls, whereas the effect was smaller with whisker stimulation (7 times). The level of α2 hypofunction induced by knockdown for priming the cortex to sensory stimulus-evoked CSDs was less efficient compared to ouabain. In line with this, K^+^ recordings during sensory stimulation disclosed that impairment in K^+^ uptake was about 4 times less after α2 knockdown compared to 0.1 mM ouabain. Contrary to the low susceptibility to stimulation-evoked CSDs, this level of α2 inhibition was sufficient to reduce the threshold of KCl-induced CSDs, suggesting that the susceptibility to CSD created by impaired extracellular K^+^ and glutamate clearance can be counteracted by the stimulation-evoked FFI limiting the intense neuronal depolarization necessary for CSD ignition, which can only be overcome with an impairment of K + and glutamate clearance larger than that produced by α2 knockdown. Similarly, priming the cortex by promoting excitation with DPCPX lowered the CSD threshold and induced spontaneous CSDs but sensory stimulation was again inefficient in triggering CSD, supporting the idea of prevention of the stimulus-evoked CSDs by FFI as discussed below. Altogether, the three different approaches used in this study (i.e. ouabain, α2 knockdown and A1 antagonism), disclose for the first time that the sensory stimulation-evoked CSDs have a highly probabilistic nature compared to KCl-triggered CSDs that are ignited by suprathreshold KCl concentrations per se, which may have been caused by concomitant activation of inhibitory activity with excitation and ensuing network oscillations during repetitive sensory stimulation. Such facilitation of CSD generation by sensory stimulation was also reported in earlier studies [25, 30], however, by employing relatively indirect approaches compared to ours. Toriumi et al. used 4–6-day long nociceptive stimulation of the trigeminal skin area and showed a threshold drop in KCl-induced CSDs in sensory cortex, whereas Bogdanov et al., exogenously applied high KCl concentrations over a widely exposed cortex to trigger CSD and observed that majority of CSDs emerged during sensory stimulation [25, 30].

### Feedforward inhibition limits CSD generation with sensory stimulation

CSD ignition requires cooperative action of K^+^ and glutamate such that whichever initially rises it is soon accompanied by an increase in the other, creating a positive feedback loop for each other’s release to the extracellular medium [31]. We envisage that, without sensory stimulation, spontaneous excitatory synaptic activity (i.e. frequent dendritic depolarizations with sporadic spiking [32]), which is prevalent in superficial cortical layers receiving dense glutamatergic corticocortical and thalamacortical input, can trigger CSD if glutamate and K^+^ cannot be rapidly removed by astrocyte processes that show reduced coverage of glutamatergic synapses in this area [33]. Therefore, partly suppressing K^+^ uptake with ouabain or promoting glutamatergic excitation by an A1R antagonist can lead to intense depolarization of dendrites, which releases further K^+^, bringing extracellular K^+^ and glutamate (supplemented by additional release from depolarized excitatory terminals until the conduction block [17, 31]) to threshold levels for triggering intense neuroglial depolarization (CSD).

For repetitive thalamocortical stimulation (visual or tactile), we hypothesized to see a synchronized depolarization of a cohort of neurons, leading to a stimulus-locked CSD when upper layers of the cortex were primed. However, with thalamocortical stimulation, FFI is also activated, limiting the thalamocortical stimulation-induced neuronal depolarization in superficial and deeper layers. Although repetitive stimulation has the potential to widen the integration window for excitation, our findings suggest that gradually attenuating FFI is still sufficient to prevent substantial depolarization [34]. Recurrent lateral inhibition may also hinder recruitment of a cohort of neurons required for CSD ignition [35]. Furthermore, extracellular adenosine building up during repetitive synaptic activity can also contribute to dampening the depolarization. In vivo, natural stimuli (e.g. light flashes we used) generally create an oscillation in gamma band (including large depolarizations) following the EPSP-IPSP due to complex interactions between feedforward and feedback connections, making the outcome of circuit behavior more probabilistic and less predictable as we observed [36]. Additionally, while the stimulus-locked excitatory activity is limited to thalamocortical synapses as the driver, spontaneous activity also encompasses dense corticortical excitatory synapses, hence, might involve more excitatory drive although less synchronized. Consequently, thalamocortical stimulation required a stronger suppression of uptake mechanisms as we saw with 0.1 mM ouabain for sufficient number of depolarized neurons to be synchronized in a brief time window and initiate the regenerative cycle culminating in CSD. That the low-concentration ouabain provided a better uptake inhibition than α2 knockdown was not only evident from K^+^ recordings (cf. Figure 5B and C) but also from the presence of ouabain-induced sporadic spontaneous CSDs. In line with our α2 knockdown findings, spontaneous CSDs have not been reported in W887R and G301R Atp1a2 knock-in or Atp1a2^tm2Kwk^ N-terminal knockout mice during experimental observation periods despite a lower KCl or electrically evoked CSD threshold [11, 12, 37], suggesting that the partial uptake insufficiency becomes significant only when challenged (e.g. with extra K^+^ or intense depolarization induced by electrical pulses).

### Pharmacological sensitization with ouabain

Ouabain is a digitalis derivative that blocks Na^+^-K^+^-ATPase ion pump on cell membranes. At concentrations that inhibit a large fraction of α1 as well as α2 and α3 isoforms of Na^+^/K^+^-ATPase in cortical and hippocampal slices in vitro, ouabain induces spreading depolarizations characterized by incomplete repolarization similar to that of anoxic depolarization [1, 9]. This shows that repolarization phase of the DC potential shift mainly reflects K^+^ reuptake and restoration of the transmembrane K^+^ gradient by Na^+^/K^+^-ATPase pump. Indeed, partial inhibition of α1-isoform in hippocampal slices allows restoration of the resting membrane potential, hence, the repolarizing phase of CSD and, leads to prolonged negative DC shifts depending on the degree of blockade of α1 subunit of Na^+^/K^+^-ATPase [9, 38]. However, lower concentrations of ouabain preferentially inhibit the α2 and α3 isoforms [9, 13, 14]. α2 is expressed on peri-synaptic astrocyte processes that play an important role in clearance of limited amounts of K^+^ and glutamate spilled from synapse, whereas neuronal isoform α3 is responsible for restoring membrane potential and Na^+^/K^+^ gradients after nerve activity [39, 40]. The CSDs we observed after topical ouabain priming had a complete and fast repolarization phase and were similar to CSDs induced by K^+^ application or pin-prick, suggesting that the concentrations of ouabain used in our study may have preferentially blocked astrocytic α2 and neuronal α3 isoforms but not significantly the α1 isoform [26], which has a thousand times lower affinity to ouabain compared to the other isoforms [41]. Imaging with fluorescent-labeled ouabain demonstrated that epidurally applied ouabain diffuses down only to the first layers of cortex that harbor dendritic tufts with a steeply decreasing concentration toward the 2^nd^-4^th^ layers where the apical dendrites generating the CSD are located, suggesting that we were able to adjust the ouabain concentration to mainly inhibit the α2/α3 isoforms in these layers (Fig. 1B). Altogether, these findings (i.e. absence of repetitive CSDs and normal CSD duration) suggest that epidural 0.1 mM ouabain application did not produce extreme perturbations, but was just enough to create the minimum conditions for CSD generation in a local cortical area as also supported by K^+^ recordings. During sensory stimulation, the fourfold rise in fluorescence from baseline, which is about 3–3.5 mM [31], corresponds to 12–14 mM K^+^; the threshold concentration to trigger CSD [42]. Similar to our approach, Calderon et al. was able to mimic rapid-onset dystonia and Parkinsonism caused by a genetic defect in α3 isoform of Na^+^/K^+^-ATPase by infusing low doses of ouabain (18 ng/h) into basal ganglia and cerebellum of the wild type mice [43]. A study on brain slices showed that rising extracellular K^+^ concentration up to 15 mM in a cortical volume of as small as 0.03–0.06 mm^3^ was sufficient to trigger CSD [42]. Such a concentration is likely to be attained within the minute volume of interstitium around synapses when K^+^ uptake during intense excitatory synaptic activity is slowed down. Under the same conditions, glutamate uptake by astrocyte processes is also suppressed as the Na^+^ gradient driving glutamate transporters is maintained by α2-Na^+^/K^+^-ATPase; thus, both proteins are intimately co-localized at the membrane of processes such that reduced expression of α2 in FHM2 W887R knock-in mice leads to decreased presence of GLT-1 at the plasma membrane [14, 44–46]. Indeed, ouabain as well as FHM2 mutations have been shown to significantly slow down the decay kinetics of glutamate signal at various stimulation intensities in the somatosensory cortex [33, 47]. Inhibition of α3 isoform with ouabain can additionally facilitate CSD induction by partially depolarizing dendrites and axon terminals [39, 48].

### *Sensitization with α2-Na*^+^*/K*^+^*-ATPase knockdown*

To specifically assess the role of α2 subunit of Na^+^/K^+^-ATPase pump in CSD susceptibility in vivo, we knocked down its expression by RNA interference, which yielded a 50% decrease in α2-Na^+^/K^+^-ATPase mRNA in the cortex. This approach indeed lowered the KCl-induced CSD threshold in line with the observations from FHM2 knock-in mice. Capuani et al. showed that defective glutamate and potassium clearance by cortical astrocytes underlies the CSD susceptibility in W887R knock-in mice due to the reduced expression of astrocytic α2 isoform of Na^+^/K^+^-ATPase as well as GLT-1 [44]. G301R FHM2 knock-in mouse had a more severe phenotype; in addition to lowered CSD threshold, their CSD duration was prolonged unlike W887R knock-in mice [12]. Unfortunately, for a fine control, the RNA interference experiments were inherently not as flexible to further lower the α2-Na^+^/K^+^-ATPase activity as was pharmacological inhibition with ouabain, which allowed disclosing the increased probability of CSD generation with sensory stimulation. Additionally, ouabain may have inhibited α3 isoform in addition to α2, hence, further slowed the K^+^ clearance as well as repolarization of dendrites and axon terminals as discussed above [39, 40]. Yet, in 1 out of 7 mice, with photic stimulation and, 1 out of 6 mice, with whisker stimulation, we were able to evoke stimulus-induced CSDs after α2 knockdown. These CSDs were likely to be triggered by stimulation as their temporal association suggested because no spontaneous CSDs were observed prior to stimulation or in 6 knockdown mice that were not stimulated but used for KCl-induced CSD threshold experiments. In line with the relatively lower stimulus-induced CSD susceptibility created by partial reduction in α2-Na^+^/K^+^-ATPase mRNA compared to the ouabain-treated group, extracellular K^+^ rise during whisker stimulation in α2-Na^+^/K^+^-ATPase knockdown mice significantly increased but to a lesser extent than that observed with 0.1 mM ouabain treatment. This is not contrary to the low KCl-induced CSD threshold detected in α2 knockdown mice, in which sub-threshold amounts of exogenously applied KCl can facilitate bringing the extracellular K^+^ levels over the threshold. A similar dependency to KCl challenge to disclose the CSD susceptibility was also observed in FHM2 knock-in mice [12] and heterozygous Atp1a2^tm1Kwk^ mice bearing a mutation at the N-terminal [37] as well as in brain slices obtained from heterozygous α2-Na^+^/K^+^-ATPase knockout mice bathed in high K^+^ medium (but not when bathed in normal ACSF or in vivo) [26]. In the absence of exogenous K^+^ challenge, the extra depolarization during stimulation caused by the relative hypofunction of α2 knockdown was generally insufficient to trigger CSD possibly due to stimulation-induced FFI as discussed above, which might have been further strengthened by reduced pace of removal of extracellular K^+^ and glutamate around excitatory synapses terminating on inhibitory neurons [16]. Unlike ouabain, α2-Na + /K + -ATPase knockdown was not limited to the superficial cortical layers because shRNA spread to all cortical layers, however, the facilitation of glutamatergic thalomocortical input in deeper layers by partial knockdown seems to have been offset by facilitation of glutamatergic input on inhibitory interneurons. The reduced K^+^ uptake during sensory stimulation-induced synaptic activity and susceptibility to CSD were not detected in blank plasmid injected control mice, ensuring that cortical injection before the experiment did not create the observed susceptibility. On the contrary, the plasmid injection-induced cortical injury may have mildly depressed whisker stimulation-evoked neural activity, hence, K^+^ release as documented after cranial window surgery [27].

Altogether, these data suggest that conditions that reduce/slow down uptake of K^+^ and glutamate released during intense sensory stimulation can increase the probability of CSD generation. In the sensory cortex, glutamate uptake by astrocyte processes slows down during sustained stimulation, which may have increased the CSD incidence during our prolonged photic or whisker stimulation [47]. It has recently been shown that migraine triggers like sleep deprivation, which reduces the availability of glycogen-derived glucose for synaptic metabolism, lowers the CSD threshold possibly because the reuptake of K^+^ and glutamate largely depends on the glycosyl units liberated from glycogen in astrocyte processes during intense/prolonged neuronal activity [49]. Insufficient fueling of α2-Na^+^/K^+^-ATPase can cause excess K^+^ accumulation during prolonged sensory stimulation as shown here in vivo [13, 44, 50]. Therefore, it is likely that migraine triggers can prime the brain for CSD generation during synaptic activity on a susceptible background by reducing capacity for reuptake of released K^+^ and glutamate.

### Sensory stimuli as migraine triggers

Migraine attacks can be triggered by a number of physiological and environmental factors. Intense sensory stimuli such as flickering or pulsating lights, bright or reflected sunshine, noise or loud sounds, strong odors are among migraine triggers [6, 7]. Bright light has been reported as a migraine trigger in 28–61% of the people with migraine with aura [6, 7, 51, 52]. Although a single trigger may set off migraine attack in some migraineurs, a number of endogenous and exogenous factors should come together in order to initiate an attack in most others. These factors, when present in combination and/or sufficient intensity, may bring a genetically/hormonally susceptible brain of migraineur up to the threshold of CSD initiation. Supporting this view, Cao et al. showed that visual stimulation of around 7 min triggered their typical headache in 8 out of 12 migraine patients [53]. In 5 of these patients, slowly propagating (at a rate of 3 to 6 mm/min) suppression of the visually evoked MRI-BOLD response in the occipital cortex was observed, in accordance with CSD. Indeed, this neuronal suppression was accompanied by MRI-BOLD changes reminiscent of the vasodilatation associated with CSD. Sensory cortices including the visual cortex may be more susceptible to CSD possibly due to relatively inefficient potassium and glutamate clearance rates or to the marginally optimal distance between astrocyte processes and excitatory synapses [25, 47]. Taken together, these findings support the hypothesis that intense synaptic activity can trigger CSD in susceptible brain of migraineurs when intrinsic mechanisms preventing CSD have been surpassed. The probabilistic nature of CSD generation in the primed mouse brain as in migraineurs points to efficient safety mechanisms that limit CSD generation even in the presence of predisposing and priming factors. The safety mechanisms are likely to be multiple, however, inhibitory interneurons and adenosine seem to play a significant role. Since adenosine derived from the ATP is a measure of intense synaptic activity, it is ideally situated to prevent uncontrolled hyperactivity and synchronization by reducing glutamate release and postsynaptic excitability in cortex as our findings suggest [54, 55]. A similar role for adenosine was also suggested for prevention of seizure activity [56]. Interestingly, antagonizing A1 subtype adenosine receptors alone was sufficient to induce spontaneous CSDs without any sensory stimulation. Under DPCPX, unregulated release of glutamate may create glutamate plumes that increase the CSD probability as shown after promoting glutamate release with veratridine [33]. Stronger dendritic depolarizations and firing caused by A1 antagonism and consequent K^+^ release may complete the conditions for CSD ignition [28]. The effect of photic stimulation could only be tested when the A1 antagonism was less efficient to spontaneously ignite CSDs. Under these conditions, sensory stimulation sporadically caused small-amplitude DC shifts coincident with suppression of EEG spikes [17]. These changes may have been produced by synchronized depolarization of a smaller aggregate of neurons than was required for a full-blown CSD, which may have been prevented to further progress by the intact glutamate and K^+^ uptake (unlike ouabain treatment), not allowing recruitment of more neurons and propagation of depolarization wave as well as by the FFI evoked with each stimulus as discussed above. Intracellular potentials recorded in vivo during light flash or whisker stimulation exhibit complex oscillations, created by integration of feedforward and feedback excitatory and inhibitory activities within a microcircuit [36]. Future studies may provide the much-needed cellular insight to the probabilistic and complex nature of CSD generation observed in our study. In this regard, increasing availability of the voltage-sensitive fluoroprobes is promising to simultaneously study the subthreshold electrical activity as well as firing of a cohort of neurons in vivo.

In conclusion, our findings suggest that normal brain is well protected against CSD generation. For CSD to be ignited under physiological conditions, several priming and predisposing factors are required as seen in migraine patients. Intense sensory stimulation has the potential to trigger CSD when co-existing conditions can bring extracellular K^+^ and glutamate concentrations over a threshold. Therefore, factors that transiently reduce uptake of K^+^ and glutamate released from synapses (e.g. inefficient fueling of α2-Na^+^/K^+^-ATPase due to reduced glycogen breakdown after sleep deprivation [57]) or facilitate glutamate release (e.g. reduced presynaptic adenosinergic inhibition) similar to mechanisms documented for FHM2 and FHM1 mutations [58], respectively, may prime the brain for CSD generation with sensory synaptic activity. However, for realization of CSD ignition, intrinsic mechanisms preventing neuronal synchronization (e.g. adenosine generated during synaptic activity) must also be overcome. Our study has important implications for future studies and was an initial step for understanding CSD generation in *non-familial common migraine*. Our findings suggest that combination of potential trigger factors such as concomitant stimulation of cholinergic arousal systems or corticocortical pathways etc., preferably in awake mice, should be investigated in future studies.

## Data Availability

The datasets used and/or analysed during the current study are available from the corresponding author on reasonable request.

## Abbreviations

aCSF: Artificial cerebrospinal fluid
Ag–AgCl: Silver-silver chloride
APG-4: Asante Potassium Green-4
ATP1A2: ATPase Na^+^/K^+^ transporting subunit alpha 2
AUC: Area under the curve
BODIPY: Boron-dipyrromethene
BOLD: Blood oxygenation level dependent
CBF: Cerebral blood flow
CCD: Charge-coupled device
cDNA: Complementary DNA
CSD: Cortical spreading depression
DC: Direct current
DMSO: Dimethyl sulfoxide
DPCPX: 8-Cyclopentyl-1,3-dipropylxanthine
FHM2: Familial hemiplegic migraine-2
GAPDH: Glyceraldehyde 3-phosphate dehydrogenase
IPG-4: ION Potassium Green-4
KCl: Potassium chloride
LED: Light emitting diode
Na^+^/K^+^-ATPase: Sodium potassium pump
qRT-PCR: Quantitative reverse transcription polymerase chain reaction
ROI: Region of interest
shRNA: Short harpin RNA
